# Delays to diagnosis among people with severe mental illness in rural Vietnam, a population-based cross-sectional survey

**DOI:** 10.1186/s12888-019-2367-1

**Published:** 2019-12-04

**Authors:** Trang Nguyen, Thach Tran, Sally Green, Arthur Hsueh, Tuan Tran, Ha Tran, Jane Fisher

**Affiliations:** 1 School of Public Health and Preventive Medicine, Monash University, Australia553 St Kilda Road, Melbourne, Victoria 3004 Australia; 2 Research and Training Centre for Community Development (RTCCD), Hanoi, Vietnam; 3 School of Population and Global Health, University of Melbourne, Melbourne, Australia

**Keywords:** Severe mental illness, Delay to diagnosis, Vietnam, Financial burden

## Abstract

**Background:**

People with severe mental illness (SMI) living in low and middle-income countries can experience extended delays to diagnosis, which hinder access to medical treatment. The aims of this study were to describe the interval to diagnosis among these people in rural Vietnam and its associated factors.

**Methods:**

A population-based cross-sectional study was conducted among people with SMI in two provinces in Vietnam. The delay to diagnosis was defined as the time between the first abnormal behaviour being observed by family members and the formal diagnosis of psychosis. A multilevel linear regression was used to examine the factors associated with the delay to diagnosis.

**Results:**

Among 404 people with SMI from 370 households, the median delay to diagnosis was 11.5 months (IQR 0–168.0). Overall, 53.7% had a delay to diagnosis of less than one year (95% CI: 48.81–58.54). The financial burden of these people on their families was nearly USD 470/year. After adjusting for other factors at individual and household levels, living in a Northern province; older age, and having psychotic diagnosis before the implementation of the National Community Mental Health program (2003) were associated with a delay of more than twelve months to diagnosis.

**Conclusions:**

These data indicate that the implementation of a national policy for community-based care has been effective in reducing the delay to diagnosis in rural Vietnam. Therefore, there is a need for strengthening the program and mental health policies, focusing on public communication to improve mental health literacy and reduce stigma against SMI.

## Background

Severe mental illness (SMI) is defined in three dimensions: clinical diagnosis, duration of the disorder, and the effect on social, family and workforce participation [1]. This term is commonly used to describe people experiencing psychotic disorders (such as schizophrenia, or bipolar, or schizoaffective disorders) which compromise their personal and social life long-term; and require care from both health and social sectors [2].

Substantial evidence suggests that prolonged delay to diagnosis is associated with worse treatment outcomes and lower likelihood of long-term recovery [3]. Therefore, identification of the onset of the first symptoms of SMI is critical to receiving an accurate diagnosis and to achieving optimal treatment outcomes [3–5]. However, many people with SMI experience a delay to diagnosis. Most studies have examined the delay to treatment for psychoses [3, 6–8]. There are limited research studies investigating nature of and reasons for the delay to diagnosis. Berk et al. recruited 240 adults with bipolar or schizoaffective disorders through public hospitals and the local print media in Melbourne and Geelong, Australia for a two-year prospective observational study (2006). The authors reported in the baseline data of this study that those who were aged 16–25 years received their first diagnosis of Bipolar I or Schizoaffective disorder around 6.5 years after their first experience of symptoms of mental illness. The delay decreased when the age of the study participants increased [9]. The delay in Berk et al.’s study included the period of having other diagnoses such as depression prior to the final diagnosis of psychotic disorders which contributed to the long delay to the ultimate diagnosis. In 2015, Patel et al. conducted a retrospective study using electronic mental health records from the South London and Maudsley NHS Foundation Trust (SLaM). The study used data about 1364 adults with bipolar disorders in the period 2007 to 2012. The delay to diagnosis was defined as the interval between the first time that participants presented to the SLaM and the time they received the diagnosis of bipolar disorders. The median delay was 62 days with a wide Inter Quartile Range from 17 to 243 days [10]. This delay is short when compared with Berk et al.’s study because it measured only the period from when these people started to seek medical support. Patel et al.’s study did not provide information about the interval from when the first psychotic symptoms were identified to medical help-seeking.

Several individual and family level factors have been associated with the delay to diagnosis among people with SMI. Spoorthy (2018) reported qualitative results of a hospital-based cross-sectional study in India of 25 people with SMI and their families. They concluded that individual level reasons such as unclear psychotic symptoms, onset coinciding with adverse life events, and impaired functioning influenced treatment seeking and interval to diagnosis. The influencing factors at household level were low mental health literacy, lack of social support, and financial constraints [11]. Other studies found that, at individual level, age and gender were associated with the delay [8, 9, 12–14]. Duration was greater among people who had first symptoms at a young age [9]. Gender was also implicated, with females being diagnosed first at a higher mean age compared to males [12]. Family-related factors such as financial burden on the family due to caring for a member with psychotic symptoms [8], lack of social support to the family [13], and lack of family awareness of the mental health problems of people with SMI and low mental health literacy [8, 13, 14] may increase the delay to diagnosis.

In comparison with high income countries, low and middle income countries are facing significant shortage of mental health professionals [15, 16], low mental health literacy, high stigma of mental illness [17] and huge treatment gap [18]. The underlying causes of these problems are the lack of mental health polices [18], low expenditure on mental health [19, 20], inappropriate organization and planning of mental health services [21], and lack of evidence based intervention and training [18].

In general, investigations of the delay to diagnosis have recruited participants from clinical facilities, not in the community or from the population at large and only limited individual and household factors have been examined. In addition, there was no consistency in the definitions of delays to diagnosis among these studies. Therefore, it may not represent the actual delay of people with SMI in the population. In order to address the gap, this study aimed to estimate the delay to diagnosis of people with SMI and its related factors at both individual and household level in a population-based sample in Vietnam.

## Methods

### Study design

The study used a population-based cross-sectional design with data collected in surveys of households with a member with SMI in rural provinces in the North and the South of Vietnam. The survey was conducted from May to June 2013.

### Setting

Vietnam’s socio-political and geographic situation is described mainly in terms of its having Northern and Southern areas. In Vietnamese history, the Northern and Southern areas were ruled by different Lords. During the periods of colonial occupation by France and the United States, Northern Vietnam was most strongly affiliated with the communist states of the Soviet Union and China. Southern Vietnam, allied with France and America developed a free market economy, and a quasi-democratic government. In 1975, the two areas were united, however, there are many differences between them in terms of culture, language-use, living standards, and common individual characteristics.

The household survey was implemented in two provinces Thanh Hoa in the North and Ben Tre in the South which are representative of the Northern and Southern areas. Thanh Hoa has the third highest population in Vietnam with approximately 3.4 million people living in 27 districts. The province has 6 coastal, 11 mountainous and 10 plain land districts and a city. The average annual income per capita was approximately 19 million Vietnam dong (about USD 980) in 2013 [22]. Ben Tre province has a population of around 1,2 million people living in 1 city and 8 districts. All are located on plain land. The average annual income per capita was nearly 26 million Vietnam dong (more than USD 1300 in 2013) [23].

Vietnam’s national community mental health program has two main activities. First, psychiatrists of provincial mental health hospitals assess whether people identified by the commune health staff or caregivers have diagnosable conditions, including schizophrenia, bipolar disorders, and epilepsy. Second, the program distributes free medications through community outreach to people meeting diagnostic criteria for one of these conditions [24]. The program was initiated in 2000, to cover more than 7000 communes in the 63 provinces in Vietnam. The program was scaled up in Thanh Hoa and Ben Tre provinces in the period 2000–2003.

### Random selection of households and sample size

A cluster sampling method was used. At national level, lists of provinces in each of the Northern and Southern areas were developed. One province was selected using simple random sampling from each list. In each province, an independent statistician chose 30 communes randomly by systematic sampling.

In each selected commune, 10 households were selected randomly from the list provided by commune health stations staff of households in which a person with a diagnosed SMI lived. People with SMI were defined as those who had been given a formal diagnosis of schizophrenia, or a bipolar affective disorder by a clinician from a public mental health hospital. They were managed by the national community mental health program at the local commune health station [24]. The household list included six households per commune for the interview, and four households to replace any of the six households if the person to be interviewed was not at home at the time the interviewers visited.

### Data collection tools and sources

Data were gathered at household and individual levels by interviewing the main caregivers. At the individual level, information about the person with SMI was collected. At the household level, a structured schedule was used to ascertain household characteristics (Table 1).

**Table 1:** Key information and data collection tools

Variable	Tool	Description
Individual level (people with SMI)		
*Primary outcome*		
Delay to diagnosis	Two questions:	The interval was measured in months. This interval also included the time period of having prior diagnoses such as depression, or anxiety.
	Time when abnormal symptoms were first noticed by family members	
	Time of the first formal diagnosis of a psychotic illness	
*Associated factors*		
Functioning scale	Adapted version of the Specific Level of Functioning Scale (SLOF)	The SLOF is a multidimensional assessment instrument which is widely used to assess people with psychotic disorders [34]. The instrument consists of six subscales and has a total of 43 items: Physical functioning (5 items), personal care skills (7 items), interpersonal relationships (7 items), social acceptability (7 items), activities (11 items), and work skills (6 items). Each item is rated using a 5-point Likert scale from 1 (poorest function) to 5 (best function). Higher scores indicate more independent functioning [35]. In this study, an adapted version of this instrument with four subscales (interpersonal relationship, social acceptability, activities and work skills) was used. This tool was translated, cultural verification, back translation and pilot tested in Vietnam.
Income	Study-specific questions of personal income of people with SMI	The personal annual income of people with SMI included the income from the paid job, monthly financial support from the government, and other sources such as donations, or gifts from relatives. The income was measured by month.
Expense	Study-specific questions of personal expenses of people with SMI	The personal annual expense of people with SMI covered all costs related to daily living (food, clothes), productivity of household members lost because of caring for people with an SMI, finding them when they wandered, compensation for any property that had been destroyed, and health service use (inpatients, and outpatient services). These costs of living expense were measured by month. The productivity loss was measured by day. Other costs were measured by year.
Household level (household having people with SMI)		
Economic status	Study specific single question about economic status	Subjective self-assessment of household heads in terms of their household economic status when compared to the local standard. There were five options: Very poor, poor, average, better off, and rich.
Household size	Number of people living in the family	Household members were defined as people living and having meals together at least 6 six months.
Social capital	Short version of the modified Adapted Social Capital Assessment Tool (SASCAT) [36]	The Short SASCAT was developed in a multi-country cohort study Young Lives. It is a quantitative tool used to measure the household social capital. The instrument consists of 20 items. This tool was validated in Vietnam and Peru with translation validity, criterion validity, and cognitive validity. It was reported as a valuable tool with known constructs and internal links among variables [36]. The response categories of yes/no.

### Procedure

Local staff of the provincial Departments of Health and of Labour, Invalids and Social Affairs were employed for data collection due to their familiarity with local accents, customs, and transport access. They were trained for three days by the research team from the Research and Training Centre for Community Development (RTCCD) in Hanoi, which was the research implementing organisation. Staff members who were qualified in terms of interpersonal skills, comprehension of the schedule, and attention to details were selected as data collectors.

Village heads invited all main caregivers of people with SMI on the lists to attend an information session at the commune health station. Information about the research and an explanation that participation was voluntary were provided to caregivers before consent to participate in an interview was sought. On the scheduled day of the interview, village heads guided the data collectors to selected households in the list.

At the household, information about the household was collected first. All caregivers were given an oral or written explanatory form of the study and were asked to sign a consent form for the collection of the household characteristics and information of people with severe mental illness by the data collectors. Those who could not write provided a thumbprint or verbal consent witnessed by the village heads. The interview about household characteristics was conducted first, then it was followed by the interview to collect the information of people with SMI. All interviews were implemented a private room to ensure the confidentiality.

The research team from RTCCD selected 5% of the completed interview schedules to re-interview caregivers using the same schedule for quality checking. All schedules were checked onsite by field supervisors for missing values and logical mistakes for correction. No name was written on the paper-based schedule.

Consent forms and completed paper-based schedules were handed to the research team at the end of the data collection day by data collectors and were stored in a locked box at the provincial Department of Labour, Invalids and Social Affair. It was returned to the RTCCD office in Hanoi city with the research team in a sealed box.

### Data management and analysis

Data from the completed paper-based schedules were manually double-entered into a password protected Access database at the RTCCD office in Hanoi. Each informant had a unique identification number that allowed the research team to distinguish individual and household characteristics. The paper records were stored in a locked cabinet and were accessible only to the research team.

Delay to diagnosis was defined as the period of time between the first symptoms of disturbed thinking or behaviour being observed by family members and the first diagnosis of a psychotic illness by a mental health specialist. As all study communes had implemented the national community mental health program by the end of 2003, comparisons were made between durations of delay to diagnoses prior to December 2003 and from January 2004 to when data were collected in 2013.

The financial burden of people with SMI on their families was calculated by deducting personal annual expenses of and costs of care from their personal annual government financial support. Among income sources of people with SMI, the monthly government financial support is a fixed and stable income. Whereas, other income sources such as income from paid jobs and donations or gifts are unstable and may change over time. All costs were collected in Vietnam dong and converted to US dollars using the exchange rate in June 2013 (1USD = 20,858 Vietnam dong). The human capital method is a widely used approach among economic studies. It measures productivity costs by estimating earnings lost due to provision of informal care [25] This method was used to estimate the productivity costs of informal care valued at USD 7.67 for eight working hours in rural Vietnam.

We used Stata, Version 13.0 to analyse the data. A *p*-value less than 0.05 was set as the level of statistical significance. The sample had two levels (households and individuals). People in the same household shared similar household characteristics. First, we conducted a descriptive analysis at individual and household levels. Second, univariate analyses were implemented to identify factors associated with duration of delay to first diagnosis and the financial burden on the families having members with mental illness. Finally, a two-level logistic regression was performed. The dependent variable (delay to diagnosis) is binary, with a value of 0 indicating the delay to diagnosis within 1 year, and a value of 1 indicating otherwise.

## Results

In total, 380 households were visited, and 370 caregivers gave consent and were included in the study. The recruitment fraction was 97%. The main reason for refusal to participate was the absence of the main caregiver from home at the time the data collectors visited. There was no difference in refusal rates between the northern and southern provinces. Overall, 370 caregivers of 404 people with SMI from 370 households provided information for the study.

### Socio-economic characteristics of people with an SMI

The socio-economic characteristics of people with an SMI and their households are presented in Table 2. Most people with an SMI in the study were described as not having completed secondary school, being cared for by family members, not being married, and having no co-morbid chronic physical health problems. Most of the households had disadvantaged and mid-level economic status; and were in rural areas.

**Table 2:** Socio-economic characteristics of people with SMI and their households in Vietnam

Variables	Thanh Hoa n (%)	Ben Tre n (%)	Total N (%)
Individual level			
Number of participants	191 (47.3)	213 (52.7)	404 (100%)
Age (Mean ± SD)	40.9 ± 16.2	41.1 ± 15.5	41.0 ± 15.8
Gender			
Male	100 (52.4)	113 (53.0)	213 (52.7)
Female	91 (47.6)	100 (47.0)	191 (47.3)
Education			
Not completed primary school	118 (61.8)	114 (53.5)	232 (57.4)
Completion of primary school	32 (16.8)	45 (21.1)	77 (19.1)
Completion of secondary school	26 (13.6)	38 (17.8)	64 (15.8)
Completion high school and higher	15 (7.8)	16 (7.6)	31 (7.7)
Receiving free antipsychotic treatment			
Yes	123 (64.4)	137 (64.3)	260 (64.4)
No	68 (35.6)	76 (35.7)	144 (35.6)
Main caregiver			
Husband/wife	60 (31.4)	19 (8.9)	79 (19.5)
Family members	127 (66.5)	175 (82.2)	302 (74.8)
Others	4 (2.1)	19 (8.9)	23 (5.7)
Marriage			
Married	68 (35.6)	25 (11.7)	93 (23.0)
Divorce/widow	21 (11.0)	24 (11.3)	45 (11.1)
Not married	102 (53.4)	164 (77.0)	266 (65.8)
Prior employment status			
Never employed	123 (64.4)	125 (58.7)	248 (61.4)
Previously employed	68 (35.6)	88 (41.3)	156 (38.6)
Having a comorbid chronic physical health problem			
Yes	52 (27.2)	48 (22.5)	100 (24.7)
No	139 (72.8)	165 (77.5)	304 (75.3)
Functioning status (Mean ± SD)	69.4 ± 20.8	86.6 ± 26.9	78.5 ± 25.7
Having psychotic diagnosis			
After 2003	97 (50.8)	129 (60.6)	226 (55.9)
Before 2003	94 (49.2)	84 (39.4)	178 (44.1)
Annual financial burden* (Mean ± SD)	442.0 ± 392.9	486.8 ± 452.3	465.6 ± 425.3
Annual income* (Mean ± SD)	262.8 ± 433.3	260.7 ± 323.6	261.7 ± 378.9
Annual Expense* (Mean ± SD)	606.8 ± 380.5	641.3 ± 450.4	625.0 ± 418.7
Household level			
Number of households	180 (48.7)	190 (51.3)	370 (100%)
Economic status			
Disadvantaged	108 (60.0)	73 (38.4)	181 (48.9)
Mid-level & advantaged	72 (40.0)	117 (61.6)	189 (51.1)
Residence			
Urban	29 (16.1)	102 (53.7)	131 (36.4)
Rural	151 (83.9)	88 (46.3)	239 (64.6)
Household size (Mean ± SD)	3.9 ± 1.5	4.0 ± 1.6	3.9 ± 1.5
Social capital (Mean ± SD)	4.1 ± 16.4	8.8 ± 35.4	6.5 ± 27.9

*Unit: US dollars

### The estimated delay to diagnosis

Among 404 study participants, the median delay to diagnosis was 11.5 months. The Inter Quartile Range was from 0 to 168.0 months. The distribution of the interval to diagnosis was skewed left. Given the date at which the National Community Mental Health program had been implemented, a binary variable was created to divide the sample into two groups: those who had been diagnosed within and those diagnosed more than one year after symptoms were first apparent. Overall 217 people (53.7, 95% Confidence Interval: 48.81–58.54) had a delay to diagnosis of up to one year, and 187 people (46.3, 95% Confidence Interval: 41.46–51.19) a delay to diagnosis of more than one year.

### The financial burden on families of care for people with SMI

The annual per capita income of people with SMI was about USD 260. The main income was from the government financial support (nearly USD 160 per year). Expenses of caring for them were more than USD 620 per year in which living costs and caregiving time contributed the most (approximately USD 500 per year). After deducting expenses from annual income, the financial burden on the families was more than USD 450 per year (Table 2).

### Factors associated with delay to diagnosis

In the univariate analyses, at individual level, participants who lived in the Northern province, had a diagnosis made before 2003, were younger, and had lower functioning scores were more likely to have been diagnosed more than a year of onset of symptoms (Table 3). At household level, there was no statistically significant association between household factors and delay to diagnosis of more than one year (Table 4).

**Table 3:** Univariate comparison of individual factors and the delay to diagnosis

Variables	Delayed duration ≤1 year n (%)	Delayed duration > 1 year n (%)	Total	*p*-value
Province				
Thanh Hoa	85 (39.2)	106 (56.7)	191 (47.3)	< 0.001
Ben Tre	132 (60.8)	81 (43.3)	213 (52.7)	
Economic status				
Disadvantaged	104 (47.9)	98 (52.4)	202 (50.0)	0.3
Mid-level & advantaged	113 (52.1)	89 (47.6)	202 (50.0)	
Gender				
Male	122 (56.2)	91 (48.7)	213 (52.7)	0.1
Female	95 (43.8)	96 (51.3)	191 (47.3)	
Education				
Not completed primary school	113 (52.1)	119 (63.6)	232 (57.4)	0.1
Completion of primary school	46 (21.2)	31 (16.6)	77 (19.1)	
Completion of secondary school	39 (18.0)	25 (13.4)	64 (15.8)	
Completion high school and higher	19 (8.7)	12 (6.4)	31 (7.7)	
Receiving free antipsychotic treatment				
Yes	140 (64.5)	120 (64.2)	260 (64.4)	0.9
No	77 (35.5)	67 (35.8)	144 (35.6)	
Main caregiver				
Husband/wife	41 (18.9)	38 (20.3)	79 (19.6)	0.3
Family members	167 (77.0)	135 (72.2)	302 (74.8)	
Others	9 (4.1)	14 (7.5)	23 (5.6)	
Marriage				
Married	49 (22.6)	44 (23.5)	93 (23.0)	0.7
Divorce/widow	22 (10.1)	23 (12.3)	45 (11.1)	
Not married	146 (67.3)	120 (64.2)	266 (65.9)	
Prior employment status				
Never employed	125 (57.6)	123 (65.8)	248 (61.4)	0.09
Previously employed	92 (42.4)	64 (34.2)	156 (38.6)	
Having a comorbid chronic physical health problem				
Yes	46 (21.2)	54 (28.9)	100 (24.8)	0.07
No	171 (78.8)	133 (71.1)	304 (75.2)	
Having psychotic diagnosis				
After 2003	160 (73.7)	66 (35.3)	226 (55.9)	< 0.001
Before 2003	57 (26.3)	121 (64.7)	178 (44.1)	
Age (mean ± SD)	39.1 ± 15.9	43.2 ± 15.4	41.0 ± 15.8	0.008
Functioning status (mean ± SD)	82.9 ± 27.1	73.4 ± 23.0	78.5 ± 25.7	0.002
Annual financial burden (mean ± SD)	462.7 ± 369.9	469.0 ± 482.7	465.6 ± 425.3	0.9

**Table 4:** Univariate comparison of household factors and the delay to diagnosis

	Households having people with delay ≤1 year	Households having people with delay > 1 year	Total	*P*-value
Economic status				
Disadvantaged	32 (53.3)	36 (58.1)	68 (55.7)	0.6
Mid-level & advantaged	28 (46.7)	26 (41.9)	54 (44.3)	
Residence				
Urban	17 (28.3)	19 (30.7)	36 (29.5)	0.7
Rural	43 (71.7)	43 (69.3)	86 (70.5)	
Household size (Mean ± SD)	1.3 ± 0.5	1.3 ± 0.6	1.3 ± 0.5	0.6
Social capital (Mean ± SD)	11.1 ± 52.4	6.1 ± 17.9	8.6 ± 38.8	0.5

In the multi-level mixed-effects logistic regression, controlling for other factors (see Table 5), only living in Ben Tre province; younger age, and having psychotic diagnosis after 2003 made significant independent contributions.

**Table 5:** Adjusted coefficient odd ratios of socio-economic characteristics and the delay to diagnosis in Vietnam

Factors	Adjusted OR	95% CI	*P*-value
Province			
Thanh Hoa (northern Vietnam)	1.00		
Ben Tre (southern Vietnam)	0.51	0.27–0.94	0.03
Residence			
Urban	1.00		
Rural	0.96	0.55–1.69	0.9
Age	1.04	1.02–1.07	< 0.001
Gender			
Male	1.00		
Female	1.1	0.67–1.78	0.7
Education			
Not completion primary school	1.00		
Completion of primary school	0.66	0.34–1.30	0.2
Completion of secondary school	0.74	(−8.02) – (−0.44)	0.4
Completion of high school and higher	0.53	(−10.58) – (− 0.14)	0.2
Functioning status	0.99	0.98–1.00	0.06
Marriage status			
Married	1.00		
Divorced/widowed	0.89	0.19–4.22	0.8
Never married	1.18	0.28–4.91	0.8
Physical comorbidities			
No	1.00		
Yes	1.49	0.85–2.63	0.2
Prior employment status			
Never employed	1.00		
Previously employed	0.57	0.30–1.07	0.08
Household economic status			
Disadvantaged	1.00		
Mid-level and advantaged	1.06	0.65–1.75	0.8
Household size	1.27	0.85–1.88	0.1
Receiving free antipsychotic treatment			
Yes	1.00		
No	1.03	0.63–1.70	0.9
Main caregiver			
Husband/wife	1.00		
*Family members*	1.34	0.31–5.83	0.6
Others	2.39	0.45–12.69	0.3
Social capital	1.00	0.99–1.01	0.9
Annual financial burden	1.00	0.99–1.00	0.7
Having psychotic diagnosis			
After 2003	1.00		
Before 2003	6.97	3.32–14.62	< 0.001

## Discussion

The major finding of this study is that the data reveal the benefit of the national policies for community-based outreach care on reducing the delay to diagnosis among people with severe mental illness in the community. While the government’s financial support contributed a major component to the income of people with SMI, which reduced the financial burden of informal care on their families, it but did not remove it.

The study had several strengths: [1] the use of multistage random sampling method from lists at national, provincial and commune levels to recruit a representative sample of caregivers of people with SMI. The recruitment fraction was high (97%), and data collection protocols were adhered to strictly by the local data collection teams. The interview schedule was carefully tested with local people prior to implementation to ensure that it was comprehensible.

However, we acknowledge some limitations. The quality of treatment in both public inpatient and outpatient mental health facilities is generally poor by international standards and characterised by human rights violations. Treatment adherence is low due to the use of old generation medication which are less effective and less well tolerated because of side effects [26]. Many people with SMI have partially or untreated symptoms. The main one being that people with SMI were not invited to contribute data about their own perspectives because when the project was designed expert advice that they were likely to be affected by chronic or acute symptoms of psychosis, to have cognitive impairments and to be unable to participate in an interview. Data about them were collected from their main caregivers. Second, telescoping bias is the recall effect in which people can perceive recent events as being more remote than they were and distant events as being more recent than they are. We acknowledge that this might have influenced estimates of the duration of the interval between recognition of symptoms and diagnosis. The estimates were based on the main caregiver’s recall and it is possible that the duration of the interval for people diagnosed a longer time ago was underestimated and that of people diagnosed more recently was over estimated because of telescoping bias. Overall, we don’t believe that this would have had a significant impact on our main finding about the impact of the National Community Mental Health program on reducing the delay to diagnosis for people with SMI. In addition, due to the limited mental health literacy of the population in Vietnam, the first experience of symptoms of mental illness may not have been recognised as requiring health care [27]. Finally, although the modified and adapted Social Capital assessment tool was validated for use in Vietnam, the SLOF was translated but had not been formally validated against a gold standard or other local comparator. We believe nevertheless that the strengths outweigh the limitations and that the results can be generalised nationally with considerable confidence.

### Delay from first symptoms of mental illness to diagnosis

In this study, the delay to diagnosis was nearly one year with a wide inter quartile range. This is higher than found by Patel et al. in London [10], but lower than reported by Berk et al. in regional Australia [9]. In addition to the difference in the definition of the delay period used and the sample recruitment method in these studies, the most important reason to explain the short delay is low mental health literacy among Vietnamese. It contributes to prevent family members from observation of first psychotic symptoms. The symptoms are noticed when they become mild/severe or dangerous to the family or the community [27].

### Financial burden of people with SMI on their family

To date, there are limited studies calculating the financial burden on the family in low and middle income countries of having members with SMI. Informal care provided by family members, relatives and friends contributes substantially to the total cost in non-health-service studies [25]. The costs are mostly attributable to productivity loss because of caregiving responsibilities for people with SMI, which preclude income generating work. In the United Kingdom (UK) (2005), these costs accounted for nearly 50% of the total discounted costs of care for people newly diagnosed with schizophrenia. The study also reported costs of £421.2 million being born by families [28]. However, due to limited information of cost estimation method, it is difficult to compare the annual expense in this study to the study in UK.

In Vietnam there is a lack of comprehensive care for people with SMI in the community such as no rehabilitation, mental health communication provided. The national community mental health program providing free medications is the only mental service in rural areas. The adherence to antipsychotic treatment of this program was reported to be low in the community [29]. The findings of this study found that the government financial support accounted for more than 60% of the income sources. Although, this support was nearly $US160 annually, it contributed significant to reduce the financial insecurity of people with SMI due to the disadvantaged household economic status. According to the Law on persons with disabilities (2010), people who are diagnosed of having severe mental illness may receive financial support from the Ministry of Labour, Invalids and Social Affairs. The financial subsidy may vary because it depends on the state budget and the severity of the mental illness [30]. The time that family members had to spend to take care of people with SMI which was mentioned as the productivity loss of informal care contributed a huge component of the expense. It is due to the low quality of treatment and the low treatment adherence of people with SMI. It led to difficult behaviours and severe disability among this group, hence their family members must spend more time of caring for them. Consequently, the average annual financial burden on the family was nearly a half of the average annual income per capita in the two provinces in 2013 (US $ 980 in Thanh Hoa and US$1300 in Ben Tre) [22, 23]. It illustrates the necessity of a comprehensive mental health care for people with SMI including rehabilitation, family education, occupational therapies, and integration activities into the community life. It would help to improve the quality of life of people with SMI and reduce the financial burden on the family.

### Associated factors of the delayed duration of diagnosis

At the individual level, we found that there were several factors related to the delay to diagnosis. First, people having the diagnosis before 2003 were more likely to delay to seek diagnosis of more than one year, 2003 is the time that all study communes implemented the national community mental health program. Therefore, this result may due to the benefit of the program. The program provided examinations to all probable cases, hence people with psychotic symptoms were more likely to receive formal diagnosis. In addition, people with schizophrenia, or bipolar disorders were provided free medications from the commune health stations, and were reviewed to receive monthly financial support from the government [29]. This encouraged caregivers to disclose and seek diagnosis for their members who had psychotic symptoms or abnormal behaviour. Therefore, the program contributed to reduce the delay to diagnosis of people with SMI in the community.

Second, participants living in the Sothern province was found to have more people with the delay to diagnosis of less than one year when comparing with those in the Northern province. This may due to the difference in economic status between the two provinces. The Northern province had 60% of participants with disadvantaged economic status, while this prevalence in the Southern province was less than 40% in this study. It is clear that poor families having members with SMI often could not afford the costs of traveling to the psychiatric hospitals and health service use. This finding is similar to the results of other studies among people with psychotic disorders that financial constraint is one of the key barriers that prevent people from seeking treatment [8, 13]. In addition, stigma and discrimination related to mental disorders, especially SMI are the common problems in low and middle income countries [31]. In Vietnam, having mental illness is not a personal problem, it also affects the honour of the family [32, 33]. Therefore, it prevents family members from seeking formal diagnosis. Southern people receive a better natural living standard and were under the long-term colonisation of American and French. Their common individual characteristics were more flexible, acceptable to a new concept. As a result, less stigma related to mental illness among Southern people when comparing with the North of Vietnam. People living in the South of Vietnam are more likely to disclose and seek diagnosis and treatment of SMI.

Finally, younger age was found to be associated positively with the group of people having the delay to diagnosis of less than one year. The result was not similar to the study in Australia among people with bipolar disorder. It found that the delayed duration decreased when the age of the study participants was increased [9]. It may be explained that in our study context, caregivers may pay more attention to younger people, then it is more likely to identify the onset of psychotic symptoms. Hence, younger may be sought diagnosis and have shorter delay. Whereas, older people may have their first symptoms long time ago, family members and caregivers may be familiar with their abnormal behaviours. It may contribute to a longer delay to diagnosis among those people.

Overall, the associated factors suggest a significant need of a communication campaign to improve mental health literacy of the community and families having a member with SMI in terms of common early symptoms, and myths of SMI. It will contribute directly to reduce the delay to diagnosis.

## Conclusion

The delay to diagnosis among people with SMI in Vietnam community was found to be associated strongly with the time receiving psychotic diagnosis. The findings suggested that the National Community Mental Health program had significant benefit in improving the delay to diagnosis of people with SMI. A need for strengthening the program to improve mental health literacy of the population is recommended.

## Data Availability

The datasets used and/or analysed during the current study are available from the corresponding author upon reasonable request.

## Abbreviations

RTCCD: Research and Training Centre for Community Development, Vietnam
SLaM: South London and Maudsley NHS Foundation Trust
SMI: Severe mental illness
UK: The United Kingdom
